# Liver Transplantation in Acute-on-Chronic Liver Failure: Excellent Outcome and Difficult Posttransplant Course

**DOI:** 10.3389/fsurg.2022.914611

**Published:** 2022-07-04

**Authors:** Guang-Hou Chen, Ruo-Lin Wu, Fan Huang, Guo-Bin Wang, Mei-Juan Zheng, Xiao-Jun Yu, Wei Wang, Liu-Jin Hou, Zheng-Hui Ye, Xing-Hua Zhang, Hong-Chuan Zhao

**Affiliations:** ^1^ Organ Transplantation Center, Department of General Surgery, The First Affiliated Hospital of Anhui Medical University, Hefei, China; ^2^ Department of Clinical Laboratory, The First Affiliated Hospital of Anhui Medical University, Hefei, China

**Keywords:** acute-on-chronic liver failure, liver transplantation, propensity score matching, single-center study, case–control studies

## Abstract

**Background:**

Acute-on-chronic liver failure (ACLF) patients have high mortality in a short period of time. This study aimed to compare the prognosis of transplanted ACLF patients to that of nontransplanted ACLF patients and decompensated cirrhosis recipients.

**Methods:**

Clinical data of 29 transplanted ACLF patients, 312 nontransplanted ACLF patients, and 60 transplanted decompensated cirrhosis patients were retrospectively collected. Propensity score matching (PSM) analysis was used to match patients between different groups.

**Results:**

After PSM, the 90-day and 1-year survival of transplanted ACLF patients was significantly longer than that of nontransplant controls. Although the 90-day survival and 1-year survival of ACLF recipients was similar to that of decompensated cirrhosis controls, ACLF recipients were found to have longer mechanical ventilation, longer intensive care unit (ICU) stay, longer hospital stay, higher incidence of tracheotomy, higher expense, and higher morbidity of complication than matched decompensated cirrhosis controls. The 90-day and 1-year survival of transplanted ACLF grade 2–3 patients was also significantly longer than that of nontransplanted controls.

**Conclusions:**

Liver transplantation can strongly improve the prognosis of ACLF patients. Despite having more burdens (including longer mechanical ventilation, longer ICU stay, higher incidence of tracheotomy, longer hospital stay, higher hospitalization expense, and higher complication morbidity), ACLF recipients can obtain similar short-term and long-term survival to decompensated cirrhosis recipients. For severe ACLF patients, liver transplantation can also significantly improve their short-term and long-term survival.

## Introduction

ACLF can develop at any stage of chronic liver disease. Under the stimulation of hepatic or extrahepatic precipitating events, patients with chronic liver disease will have a rapid deterioration of liver function, leading to high mortality in a short period of time (1). A retrospective cohort study of patients from the United States showed the 28-day and 90-day mortality rates for nontransplanted ACLF patients defined by the Asian Pacific Association for the Study of the Liver (APASL) were, respectively, 41.9% and 56.1% and for nontransplanted ACLF patients defined by the European Association for the Study of the Liver-Chronic Liver Failure (EASL-CLIF) Consortium were respectively 37.6% and 50.4% (2). HBV-ACLF patients prospectively collected from the APASL-ACLF Research Consortium and the Chinese Study Group showed that 28-day and 90-day transplantation-free mortality rates were, respectively, 27.8% and 40.0% (3).

Studies have found that liver transplantation is an important rescue therapy for ACLF patients (4–9), but some problems remain disputable. For example, O’Leary et al. reported that patients with and without ACLF had similar survival after transplantation (4), but studies by Levesque et al. (5) and Huebener et al. (9) showed that ACLF patients have higher mortality after transplantation compared to those transplanted without ACLF. In addition, the criteria of ACLF for most published studies were from the EASL-CLIF Consortium, with few reports on the diagnosis and treatment of ACLF based on APASL criteria. In this study, we reported the outcome of transplanted ACLF patients [according to the definition of ACLF in the APASL (2019) guideline] and compared the prognosis of these patients to that of nontransplanted ACLF patients and patients transplanted with decompensated cirrhosis.

## Patients and Methods

### Patients

We analyzed the clinical data of all liver transplantation recipients and all nontransplanted ACLF patients in the First Affiliated Hospital of Anhui Medical University between January 1, 2015, and July 31, 2021. Transplanted ACLF patients were retrospectively included if they fulfilled the following inclusion criteria: age >18 years old, liver transplantation for ACLF defined by the APASL (2019) guideline, and with a direct intrahepatic precipitating event. Exclusion criteria were multiple organ transplantation, liver transplantation for fulminant hepatic failure, patients with prior history of acute decompensation, and patients with an extrahepatic precipitating event. All of these included patients underwent deceased donor liver transplantation, and the ABO blood group was compatible. This study was approved by the Clinical Medicine Research Ethics Committee of the First Affiliated Hospital of Anhui Medical University, and informed consent was obtained from each patient included in the study.

Donor variables include donor age, body mass index (BMI), warm ischemia time, cold ischemia time, and steatosis of the graft biopsy. Recipient data were collected at the time of admission, at transplantation, and after transplantation. The following variables were collected at admission: gender, age, the etiology of chronic liver disease, precipitating events of ACLF, laboratory data [including total bilirubin, the international normalized ratio (INR), and creatinine], the Asian Pacific Association for the Study of the liver acute-on-chronic liver failure research consortium (AARC) score, the Child–Pugh score, the model for end-stage liver disease (MELD) score, and plasma exchange therapy. The following variables were collected at transplantation: anhepatic phase and intraoperative red blood cell (RBC) transfusion. The following variables were collected after transplantation: length of ICU stay, length of stay in the hospital, length of vasoactive drug use, length of mechanical ventilation, length of continuous renal replacement therapy (CRRT), hospitalization expense, short-term complications (90-day), and long-term survival (1-year).

Clinical data of nontransplanted ACLF patients were collected, including gender, age, the etiology of chronic liver disease, precipitating events of ACLF, laboratory data (including total bilirubin, INR, and creatinine), the AARC score, the MELD score, short-term survival (90-day), and long-term survival (1-year).

### Statistical Analysis

The categorical variables were expressed as frequency and percentage. In the case of normal distribution, the quantitative variables were expressed as the mean (standard deviation) or the median (interquartile range). The paired Student’s *t*-test was used to compare the MELD and AARC scores of ACLF patients at admission and transplantation. The Kaplan–Meier method was used to estimate the survival curves. The 90-day and 1-year survival rates were calculated with a 95% confidence interval and were compared using the log-rank test. The hazard ratio (HR) of mortality was obtained by the Cox regression model. A logistic regression model was used to perform a univariate analysis of the main characteristics related to pulmonary infection. Comparisons of the patients’ quantitative variables were performed using the Mann–Whitney *U* test. Fisher’s exact test was used to compare the frequency of categorical variables between groups. Propensity score matching analysis was used to match the patients between different groups. A statistical test was performed on the two-tailed *α* level of 0.05. Data were analyzed using the SPSS 26.0 software package and GraphPad prism 9.0.

## Results

Between January 1, 2015, and July 31, 2021, 125 patients received liver transplantation in the First Affiliated Hospital of Anhui Medical University, including 29 patients (23.2%) transplanted with ACLF and 60 patients (46.5%) transplanted with decompensated cirrhosis. A total of 312 nontransplanted ACLF patients were treated in the Department of Hepatology in the First Affiliated Hospital of Anhui Medical University during this period.

### Short-Term Case–Control Study of Transplanted and Nontransplanted ACLF Patients

**Figure 1:**
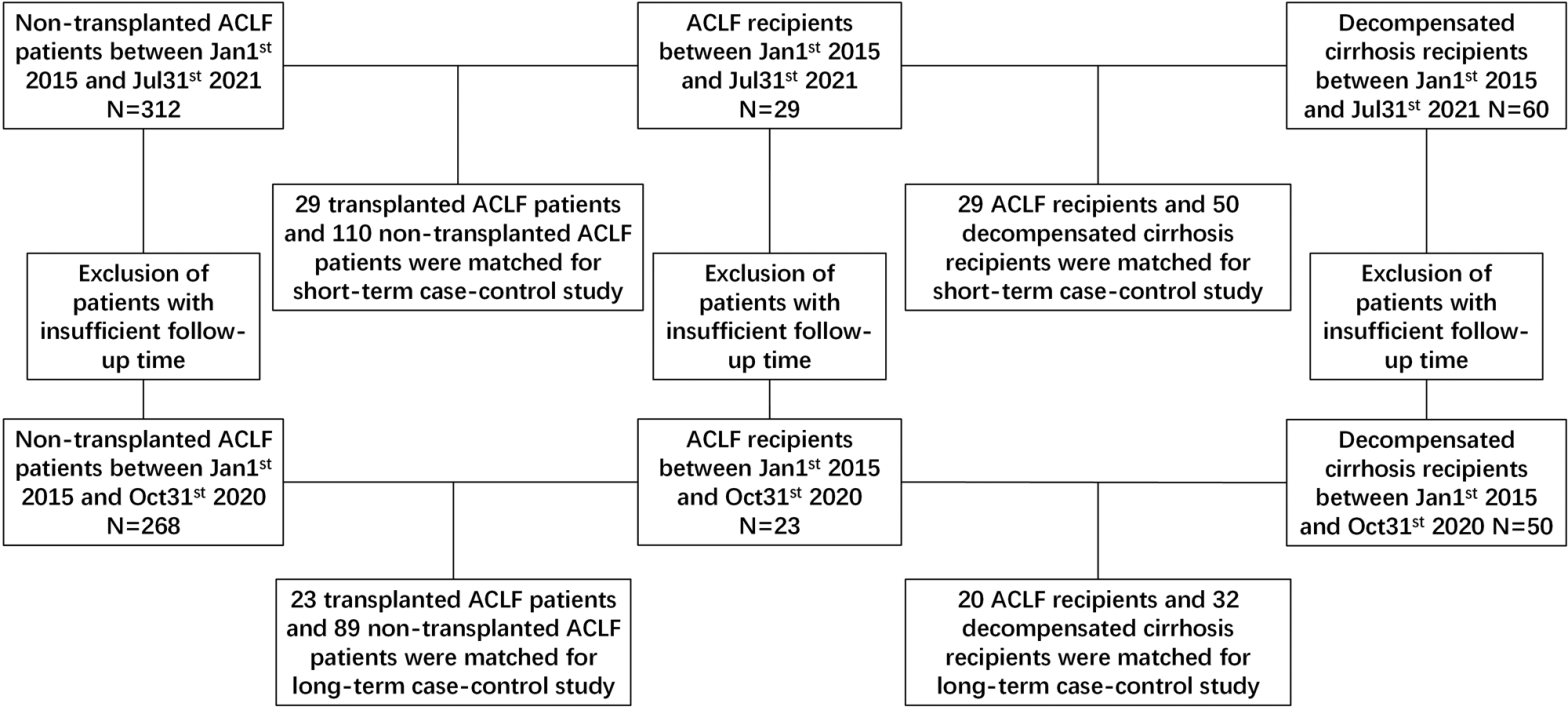
Flowchart illustrates the patients identified in this study.

Transplanted ACLF patients and nontransplanted ACLF patients were matched by PSM (transplanted:nontransplanted = 1:4) based on the gender, age, MELD score, and AARC score at admission (Figure 1). A total of 110 nontransplanted ACLF patients were matched with 29 transplanted ACLF patients. After PSM, there was no difference in the baseline data between these two groups of patients (Supplementary Table 1).

**Figure 2:**
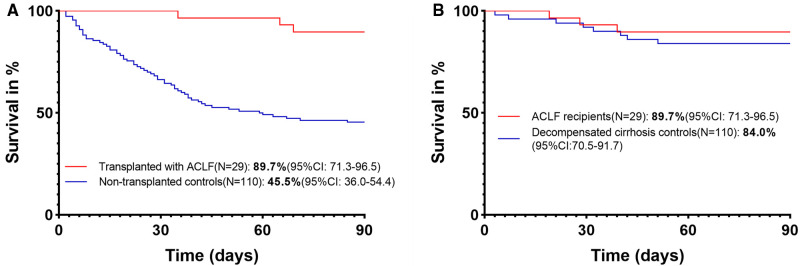
(**A**) 90-day survival of transplanted and nontransplanted ACLF patients. (**B**) 90-day survival of ACLF and decompensated cirrhosis recipients.

All of these transplanted ACLF patients were first treated with conservative treatment in the Department of Hepatology before transplantation. Liver failure of these ACLF patients further progressed after conservative treatment, with an increase of MELD score (27[25–32] vs. 25[22–29], *P* = 0.006) and AARC score (10[8–10] vs. 8[7–8], *P* < 0.0001). The most common etiology of chronic liver disease is hepatitis B, and the most common precipitating event for ACLF was HBV reactivation (Table 1). There was no difference in laboratory data, MELD score, and AARC score at admission between matched transplanted and nontransplanted ACLF patients (Table 1). The 90-day survival rate of transplanted ACLF patients was significantly higher than that of matched nontransplanted controls: 89.7% (95% CI, 71.3–96.5) vs. 45.5% (95% CI, 36.0–54.4) with a hazard ratio (HR) for mortality of 0.13 (95% CI, 0.08–0.23, *P* < 0.0001) (Figure 2A).

**Table 1:** Main characteristics of transplanted and nontransplanted ACLF patients.

Characteristics	Transplanted ACLF patients (*N* = 29)	Nontransplanted ACLF patients (*N* = 110)	*P*-value
Age (years)	45(42–53)	46.5(38–54)	0.90
Gender (M/F)	21/8	85/25	0.63
Etiology of chronic liver disease			
Hepatitis B	18(62.1%)	86(78.1%)	0.37
Alcoholic	6(20.7%)	14(12.7%)	0.37
Others	5(17.3%)	10(9.1%)	
Precipitating events			
HBV reactivation	13(44.8%)	47(42.7%)	0.84
Hepatotoxic drugs	4(13.8%)	20(18.2%)	0.78
Hepatic insult by infection	4(13.8%)	30(27.2%)	0.15
Others	8(27.6%)	13(11.8%)	
Total bilirubin (mmol/L)	235.2(216.9–349.4)	262.3(183.4–375.2)	0.76
INR	2.11(1.97–2.42)	2.05(1.82–2.57)	0.44
Creatinine (µmol/L)	65.3(53.0–83.0)	64(52.1–78.6)	0.79
MELD score at admission	25(22–29)	25(23–28)	0.97
AARC score at admission	8(7–8)	8(7–8)	0.63
90-day survival (%)	89.7(95% CI, 71.3–96.5)	45.5(95% CI, 36.0–54.4)	<0.0001

### Short-Term Case–Control Study of ACLF Recipients and Decompensated Cirrhosis Recipients

A total of 29 ACLF recipients and 50 decompensated cirrhosis recipients were matched by PSM (ACLF recipients:decompensated cirrhosis recipients = 1:2) based on gender, age, donor age, donor BMI, warm ischemia time, cold ischemia time, anhepatic phase, and RBC transfusion (Figure 1). After PSM, there was no difference in the baseline data between these two groups of patients (Supplementary Table 1).

The MELD score and Child–Pugh score at transplantation of ACLF recipients were significantly higher than those of matched decompensated cirrhosis recipients (Table 2). ACLF recipients were found to have longer mechanical ventilation, longer ICU stay, longer hospital stay, higher incidence of tracheotomy, and higher expenses than matched decompensated cirrhosis recipients (Table 2). ACLF recipients have higher morbidity of complication (particularly for pulmonary infection) compared to decompensated cirrhosis controls (Table 2). Since the pulmonary infection was found to be the most common complication of ACLF recipients in this study, we further analyzed the potential independent predictors of pulmonary infection. ICU stay ≥67 h (median cutoff value) was found to be associated with pulmonary infection (Table 3).

**Table 2:** Main characteristics of ACLF and decompensated cirrhosis recipients.

Characteristics	ACLF recipients (*N* = 29)	Decompensated cirrhosis recipients (*N* = 50)	*P*-value
Age (years)	45(42–53)	48(36.5–54.75)	0.49
Gender (M/F)	21/8	36/14	0.99
Etiology of liver disease			
Hepatitis B	18(62.1%)	27(54.0%)	0.64
Alcoholic	6(20.7%)	6(12.0%)	0.19
Others	5(17.3%)	17(34.0%)	
MELD score	27(25–32)	14.5(11–17)	<0.0001
Child–Pugh score	11(11–12)	9(7–10)	<0.0001
Donor age (years)	52(42–54)	49(35.8–57)	0.90
Donor BMI (kg/m^2^)	23.9(22.5–24.8)	23(21.9–24.2)	0.23
Graft steatosis	12(41.4%)	17(34.0%)	0.63
Warm ischemia time (min)	22(18–24)	20(15–24.75)	0.43
Cold ischemia time (min)	337.5(269.5–428.75)	333.5(285.25–469)	0.64
Anhepatic phase (min)	51(45–59)	54(49.3–61)	0.20
Red blood cell transfusion (U)	6(4–8)	5.75(3–8)	0.22
Vasoactive drug (h)	6(0–35)	6(0–12.375)	0.53
Mechanical Ventilation (h)	28(20–188)	16(16–31)	0.0022
CRRT	5(17.2%)	3(6.0%)	0.14
Tracheotomy	9(31.0%)	4(8.0%)	0.004
ICU stay (h)	67(50–279)	47.5(32.25–65.25)	0.0029
Length of stay in hospital (days)	34(24–43)	23(18–29.5)	0.0031
Expense (RMB)	322,381(227, 876–478,550)	181,180(154, 542–225,794)	<0.0001
Complications (%)	26(89.7%)	27(54.0%)	0.0012
Pulmonary infection (%)	23(79.3%)	17(34.0%)	0.0001
90-day survival (%)	89.7% (95% CI, 68.3–96.5)	84.0% (95% CI, 70.5–91.7)	0.50

**Table 3:** Independent predictors of pulmonary infection of ACLF recipients.

Characteristics	Hazard ratio	95% CI	*P*-value
Age ≥45 years^a^	0.28	0.04–1.76	0.17
AARC ≥10^a^	1.33	0.24–7.40	0.74
MELD ≥27^a^	0.92	0.17–5.16	0.93
Cold ischemia time ≥337.5 min^a^	0.75	0.14–4.17	0.74
Red blood cell transfusion ≥6 U^a^	0.33	0.05–2.10	0.24
Vasoactive drug use ≥6 h^a^	1.93	0.34–10.78	0.46
Mechanical ventilation ≥28 h^a^	0.92	0.17–5.16	0.93
ICU stay ≥67 h^a^	0.10	0.01–0.94	0.04

^a^ *Median cutoff value*.

The 90-day survival rate of ACLF recipients was 89.7% (95% CI, 71.3–96.5), which was similar to that of decompensated cirrhosis controls (84.0%[95% CI, 70.5–91.7]) with an HR for mortality of 0.66 (95% CI, 0.19–2.23, *P* = 0.50) (Figure 2B). These results suggest that ACLF recipients can obtain similar short-term survival to decompensated cirrhosis recipients.

### Long-Term Case–Control Study Between Transplanted ACLF Patients and Nontransplanted ACLF Patients, Decompensated Cirrhosis Recipients.

A total of 216 nontransplanted ACLF patients, 23 transplanted ACLF patients, and 50 decompensated cirrhosis recipients were included in this long-term case–control study after the exclusion of patients with insufficient follow-up time (Figure 1).

**Figure 3:**
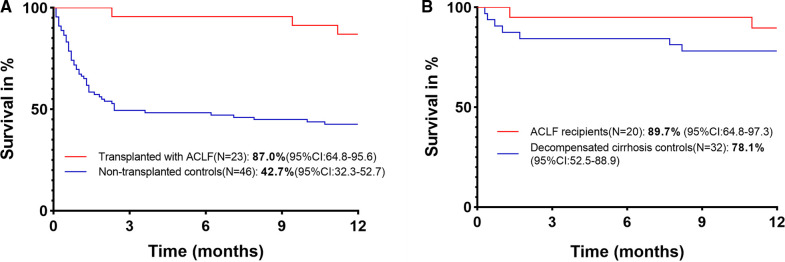
(**A**) 1-year survival of transplanted and nontransplanted ACLF patients. (**B**) 1-year survival of ACLF and decompensated cirrhosis recipients.

Transplanted ACLF patients and nontransplanted ACLF patients were matched based on gender, age, MELD score, and AARC score at admission, with PSM (transplanted:nontransplanted = 1:4) (Figure 1). A total of 89 nontransplanted ACLF patients were matched with 23 transplanted ACLF patients. After PSM, there was no difference in the baseline data between these two groups of patients (Supplementary Table 2). The 1-year survival rate of transplanted ACLF patients was 87.0% (95% CI, 64.8–95.6), which was also significantly higher than that of nontransplanted controls (42.7%[95% CI, 32.3–52.7]) with an HR for mortality of 0.16 (95% CI, 0.09–0.27, *P* = 0.0003) (Figure 3A).

A total of 20 ACLF recipients and 32 decompensated cirrhosis recipients were matched by PSM (ACLF recipients:decompensated cirrhosis recipients = 1:2) based on gender, age, donor age, donor BMI, warm ischemia time, cold ischemia time, anhepatic phase, and RBC transfusion. After PSM, no baseline data difference was found in these two groups of patients (Supplementary Table 2). The 1-year survival rate of ACLF recipients was 89.7% (95% CI, 64.8–97.3), which was similar to that of decompensated cirrhosis controls (78.1% [95% CI, 52.5–88.9]) with an HR for mortality of 0.42 (95% CI, 0.11–1.60, *P* = 0.27) (Figure 3B).

### Short-Term and Long-Term Case–Control Study of Transplanted and Nontransplanted Severe ACLF Patients

The current era of organ shortage requires careful selection of severe ACLF recipients, which is mandatory to limit the risk of futile liver transplantation. ACLF grade 2–3 patients were screened out specifically according to the AARC score at admission to analyze the therapeutic effect of liver transplantation on severe ACLF patients. A total of 18 transplanted ACLF grade 2–3 patients and 186 nontransplanted ACLF grade 2–3 patients were included in the study of the short-term survival. A total of 14 transplanted ACLF patients and 162 nontransplanted ACLF patients were included in the study of the long-term survival. Patients were matched by PSM (transplanted:nontransplanted = 1:4) based on gender, age, MELD score, and AARC score at admission (Supplementary Table 3).

After PSM, the 90-day survival rate of transplanted ACLF grade 2–3 patients was significantly higher than that of matched nontransplanted controls: 88.9% (95% CI, 62.8–97.1) vs. 33.3% (95% CI, 22.6–44.4) with an HR for mortality of 0.11 (95% CI, 0.06–0.20, *P* < 0.0001) (Supplementary Figure 1A). After PSM, the 1-year survival rate of transplanted ACLF grade 2–3 patients was 92.9% (95% CI, 59.1–99.0), which was also higher than that of matched nontransplanted controls (28.0%[95% CI, 16.4–40.7]) with an HR for mortality of 0.06 (95% CI, 0.03–0.12, *P* < 0.0001) (Supplementary Figure 1B). These results suggest that liver transplantation can also strongly improve the short-term and long-term survival of severe ACLF patients.

## Discussion

This study applied the APASL (2019) guideline to explore the short-term and long-term prognosis of ACLF recipients in a cohort of Chinese patients. PSM was used to match the patients from different groups. Several findings were reported in this study. First, our findings corroborate the result that liver transplantation can significantly improve the short-term and long-term survival of ACLF patients. Second, although previously published studies have reported that ACLF patients have higher 90-day mortality compared to patients transplanted without ACLF (5), ACLF recipients in this study were found to have similar short-term and long-term survival compared to decompensated cirrhosis recipients. However, more burdens were found in ACLF recipients than in matched decompensated cirrhosis controls, including higher complication morbidity, longer mechanical ventilation, higher incidence of tracheotomy, longer ICU stay, longer hospital stay, and higher expenses. Third, pulmonary infection was found to be the most common complication in transplanted ACLF patients, and prolonged ICU stay was found to be an independent predictor of pulmonary infection. In addition, short-term and long-term survival of severe ACLF patients can also be strongly improved by liver transplantation. Although our study validated some conclusions drawn by previous studies, we still reached plenty of innovative conclusions, which can provide a valuable reference for the follow-up work of liver transplantation in the treatment of ACLF patients.

The ACLF patients in this study were defined by the APASL (2019) guideline. The APASL defined ACLF as an acute hepatic insult manifesting as jaundice (serum bilirubin ≥5 mg/dl (85 mmol/L) and coagulopathy (INR ≥ 1.5 or prothrombin activity <40%) complicated within 4 weeks by clinical ascites and/or encephalopathy in a patient with previously diagnosed or undiagnosed chronic liver disease/cirrhosis. The main positive criteria are a precipitating event that has a direct effect on the liver and acute hepatic insult that causes acute liver failure (10). The main negative criteria are no prior history of acute decompensation in patients with cirrhosis and no extrahepatic precipitating event. The EASL-CLIF consortium defined ACLF based on the CLIF-C organ failure (CLIF-C OF) scoring system that assesses six organ systems (liver, kidney, brain, coagulation, circulation, and respiration) (11), and renal failure was considered as a necessary condition for the diagnosis ACLF (12). Differences between APASL and EASL-CLIF Consortium ACLF definitions are due to not only the consequences of the distinct types of underlying liver disease and precipitating events in different geographic regions but also the distinct objectives by which both definitions were designed (1, 13). EASL-CLIF Consortium's definition was to characterize a syndrome in which organ failure is considered a central part of this syndrome, while the APASL definition mainly emphasizes the recognition of ACLF patients. ACLF patients in this study were defined according to the APASL (2019) guideline, which emphasizes precipitating event that has a direct effect on the liver. Precipitating events of ACLF in this study include HBV reactivation, hepatotoxic drugs, hepatic insult by infection, alcoholic hepatitis, liver surgery, and nonidentifiable precipitating events. Regardless of the type of precipitating events, these ACLF patients first showed hepatic insult with liver failure, which we identified as direct intrahepatic damage.

Pulmonary infection is a common cause of mortality in liver transplantation recipients (14). In this study, pulmonary infection was found to be the most common complication of ACLF recipients during hospitalization. Several characteristics were analyzed to be potential independent predictors for pulmonary infection, and ICU stay ≥67 h (median cutoff value) was found to be associated with pulmonary infection. These results suggest that ACLF recipients were more likely to develop a pulmonary infection due to prolonged ICU stay.

The severity of nontransplanted ACLF was thought to be positively correlated with short-term mortality (15, 16). Death occurred rapidly in nontransplanted severe ACLF patients. The 90-day survival rate of matched nontransplanted ACLF grade 2–3 patients was only 33.3%. With liver transplantation, the 90-day survival rate of ACLF grade 2–3 patients can be strongly improved from 33.3% to 88.9% (Supplementary Figure 1). Nevertheless, only a small proportion of ACLF patients (8.5% in this study) have the opportunity to receive liver transplantation, which means that most ACLF patients were not selected for transplantation (6, 17). Liver transplantation should be discussed early in ACLF patients (especially in severe ACLF patients).

In contrast with former studies showing increased mortality in severe ACLF recipients (7, 8, 18), the short-term and long-term survival was found to be similar between ACLF and decompensated cirrhosis recipients in this study. It may be due to the heterogeneity of the patient population and the different ACLF definitions. ACLF recipients were found to have higher complication morbidity, which leads to longer mechanical ventilation, higher incidence of tracheotomy, longer ICU stay, longer hospital stay, and higher hospitalization expense. Therefore, repeated systematic screening for infection and careful monitoring was needed for ACLF recipients.

There are several limitations to our study. We compared the outcome of ACLF recipients to that of non-ACLF recipients, but the outcome of different ACLF grade patients has not been discussed due to the limited ACLF recipients included in this study. Furthermore, several characteristics were found to be associated with post-LT mortality in formerly published studies (19), while no characteristic was found to be associated with mortality of transplanted ACLF patients in this study.

In conclusion, treatment of ACLF requires the participation of multiple departments, including the Department of Hepatology, Department of Liver Transplantation, and ICU. ACLF patients treated in the Department of Hepatology should be listed for LT as soon as conservative treatment does not work. Rapid evaluation and smooth surgical procedures are needed in the Department of Liver Transplantation. Repeated systematic screening for infection and careful monitoring are needed for ACLF recipients in ICU after transplantation. With the cooperation of multiple departments, the survival of ACLF patients can be strongly improved.

## Data Availability

The original contributions presented in the study are included in the article/Supplementary Material, further inquiries can be directed to the corresponding author.

## References

[1] Arroyo V Moreau R Kamath PS Jalan R Ginès P Nevens F Acute-on-chronic liver failure in cirrhosis. Nat Rev Dis Primers. (2016) 2:16041. 10.1038/nrdp.2016.41 27277335

[2] Mahmud N Kaplan DE Taddei TH Goldberg DS. Incidence and mortality of acute-on-chronic liver failure using two definitions in patients with compensated cirrhosis. Hepatology (Baltimore, MD). (2019) 69(5):2150–63. 10.1002/hep.30494 30615211 PMC6461492

[3] Chen T Yang Z Choudhury AK Al Mahtab M Li J Chen Y Complications constitute a major risk factor for mortality in hepatitis B virus-related acute-on-chronic liver failure patients: a multi-national study from the Asia-Pacific region. Hepatol Int. (2019) 13(6):695–705. 10.1007/s12072-019-09992-x 31650510

[4] O’Leary JG Bajaj JS Tandon P Biggins SW Wong F Kamath PS Outcomes after listing for liver transplant in patients with acute-on-chronic liver failure: the multicenter North American consortium for the study of end-stage liver disease experience. Liver Transplant. (2019) 25(4):571–9. 10.1002/lt.25426 PMC11075742 30724010

[5] Levesque E Winter A Noorah Z Daurès JP Landais P Feray C Impact of acute-on-chronic liver failure on 90-day mortality following a first liver transplantation. Liver Int. (2017) 37(5):684–93. 10.1111/liv.13355 28052486

[6] Artru F Louvet A Ruiz I Levesque E Labreuche J Ursic-Bedoya J Liver transplantation in the most severely ill cirrhotic patients: a multicenter study in acute-on-chronic liver failure grade 3. J Hepatol. (2017) 67(4):708–15. 10.1016/j.jhep.2017.06.009 28645736

[7] Thuluvath PJ Thuluvath AJ Hanish S Savva Y. Liver transplantation in patients with multiple organ failures: feasibility and outcomes. J Hepatol. (2018) 69(5):1047–56. 10.1016/j.jhep.2018.07.007 30071241

[8] Sundaram V Jalan R Wu T Volk ML Asrani SK Klein AS Factors associated with survival of patients with severe acute-on-chronic liver failure before and after liver transplantation. Gastroenterology. (2019) 156(5):1381–91.e3. 10.1053/j.gastro.2018.12.007 30576643

[9] Huebener P Sterneck MR Bangert K Drolz A Lohse AW Kluge S Stabilisation of acute-on-chronic liver failure patients before liver transplantation predicts post-transplant survival. Aliment Pharmacol Ther. (2018) 47(11):1502–10. 10.1111/apt.14627 29611203

[10] Sarin SK Choudhury A Sharma MK Maiwall R Al Mahtab M Rahman S Acute-on-chronic liver failure: consensus recommendations of the Asian Pacific association for the study of the liver (APASL): an update. Hepatol Int. (2019) 13(4):353–90. 10.1007/s12072-019-09946-3 31172417 PMC6728300

[11] Moreau R Jalan R Gines P Pavesi M Angeli P Cordoba J Acute-on-chronic liver failure is a distinct syndrome that develops in patients with acute decompensation of cirrhosis. Gastroenterology. (2013) 144(7):1426–37, 1437.e1-9. 10.1053/j.gastro.2013.02.042 23474284

[12] Moreau R Gao B Papp M Bañares R Kamath PS. Acute-on-chronic liver failure: a distinct clinical syndrome. J Hepatol. (2021) 75(Suppl 1):S27–S35. 10.1016/j.jhep.2020.11.047 34039489

[13] Aday A O’Leary JG. Acute on chronic liver failure: definition and implications. Clin Liver Dis. (2020) 24(3):521–34. 10.1016/j.cld.2020.04.004 32620286

[14] De Gasperi A Feltracco P Ceravola E Mazza E. Pulmonary complications in patients receiving a solid-organ transplant. Curr Opin Crit Care. (2014) 20(4):411–9. 10.1097/mcc.0000000000000120 24979712

[15] Hernaez R Liu Y Kramer JR Rana A El-Serag HB Kanwal F. Model for end-stage liver disease-sodium underestimates 90-day mortality risk in patients with acute-on-chronic liver failure. J Hepatol. (2020) 73(6):1425–33. 10.1016/j.jhep.2020.06.005 32531416 PMC10424237

[16] Sundaram V Shah P Wong RJ Karvellas CJ Fortune BE Mahmud N Patients with acute on chronic liver failure grade 3 have greater 14-day waitlist mortality than status-1a patients. Hepatology (Baltimore, MD). (2019) 70(1):334–45. 10.1002/hep.30624 30908660 PMC6597310

[17] Levesque E Saliba F Ichaï P Samuel D. Outcome of patients with cirrhosis requiring mechanical ventilation in ICU. J Hepatol. (2014) 60(3):570–8. 10.1016/j.jhep.2013.11.012 24280294

[18] Abdallah MA Waleed M Bell MG Nelson M Wong R Sundaram V Systematic review with meta-analysis: liver transplant provides survival benefit in patients with acute on chronic liver failure. Aliment Pharmacol Ther. (2020) 52(2):222–32. 10.1111/apt.15793 32490550

[19] Sundaram V Kogachi S Wong RJ Karvellas CJ Fortune BE Mahmud N Effect of the clinical course of acute-on-chronic liver failure prior to liver transplantation on post-transplant survival. J Hepatol. (2020) 72(3):481–8. 10.1016/j.jhep.2019.10.013 31669304 PMC7183313

