# Supplementary material for: Liver Transplantation in Acute-on-Chronic Liver Failure: Excellent Outcome and Difficult Posttransplant Course
Source: Front Surg. 2022 Jul 4;9:914611. doi: 10.3389/fsurg.2022.914611 (PMC9289224; doi:10.3389/fsurg.2022.914611)
Supplement: Supplementary file 1 [file Table_1_v1.docx]

Supplementary Table 1: Propensity score matching of short-term case-control study

|  | **Before propensity score matching** | | | **After propensity score matching** | | |
| --- | --- | --- | --- | --- | --- | --- |
| **Characteristics** | **Transplanted ACLF (N=29)** | **Non-transplanted ACLF (N=312)** | **P value** | **Transplanted ACLF (N=29)** | **Non-transplanted ACLF (N=110)** | **P value** |
| Age (y) | 45(42-53) | 48(40-57) | 0.53 | 45(42-53) | 46.5(38-54) | 0.90 |
| Gender (M/F) | 21/8 | 251/61 | 0.33 | 21/8 | 85/25 | 0.63 |
| AARC score | 8 (7-8) | 8(7-9) | 0.85 | 8 (7-8) | 25(23-28) | 0.97 |
| MELD score | 25 (22-29) | 26(23-31) | 0.13 | 25 (22-29) | 8 (7-8) | 0.63 |
| **Characteristics** | **ACLF recipients (N=29)** | **Decompensated cirrhosis recipients (N=60)** | **P value** | **ACLF recipients (N=29)** | **Decompensated cirrhosis recipients (N=50)** | **P value** |
| Age (y) | 45(42-53) | 48(37.75-54) | 0.57 | 45(42-53) | 48(36.5-54.75) | 0.49 |
| Gender | 21/8 | 42/18 | 0.99 | 21/8 | 36/14 | 0.99 |
| Donor age (y) | 52(42-54) | 49(34-57) | 0.87 | 52(42-54) | 49(35.8-57) | 0.90 |
| Donor BMI (kg/m^2^) | 23.9(22.5-24.8) | 23.2(22.0-24.2) | 0.30 | 23.9(22.5-24.8) | 23(21.9-24.2) | 0.23 |
| WIT (mins) | 22(18-24) | 19(14-24) | 0.18 | 22(18-24) | 20(15-24.75) | 0.43 |
| CIT (mins) | 337.5(269.5-428.75) | 352(293.25-491.75) | 0.41 | 337.5(269.5-428.75) | 333.5(285.25-469) | 0.64 |
| Anhepatic phase (mins) | 51(45-59) | 56(50-63) | 0.02 | 51(45-59) | 54(49.3-61) | 0.20 |
| RBC (U) | 6(4-8) | 6(4-9.25) | 0.50 | 6(4-8) | 5.75(3-8) | 0.22 |

WIT: warm ischemia time, CIT: cold ischemia time, AHT: anhepatic time, RBC: red blood cell.
