# Supplementary material for: Liver Transplantation in Acute-on-Chronic Liver Failure: Excellent Outcome and Difficult Posttransplant Course
Source: Front Surg. 2022 Jul 4;9:914611. doi: 10.3389/fsurg.2022.914611 (PMC9289224; doi:10.3389/fsurg.2022.914611)
Supplement: Supplementary file 2 [file Table_2_v1.docx]

Supplementary Table2: Propensity score matching of long-term case-control study

|  | **Before propensity score matching** | | | **After propensity score matching** | | |
| --- | --- | --- | --- | --- | --- | --- |
| **Characteristics** | **Transplanted ACLF (N=23)** | **Non-transplanted ACLF (N=268)** | **P value** | **Transplanted ACLF (N=23)** | **Non-transplanted ACLF (N=89)** | **P value** |
| Age (y) | 44(42-52.5) | 48(40-57) | 0.43 | 44(42-52.5) | 45(36-54) | 0.76 |
| Gender(M/F) | 17/6 | 214/54 | 0.04 | 17/6 | 63/26 | 0.99 |
| AARC score | 8(7-8) | 8(7-9) | 0.88 | 8(7-8) | 8(7-9) | 0.90 |
| MELD score | 25(22.5-27) | 26(24-31.25) | 0.14 | 25(22.5-27) | 26(23-29) | 0.64 |
| **Characteristics** | **ACLF recipients (N=23)** | **Decompensated cirrhosis recipients (N=50)** | **P value** | **ACLF recipients (N=20)** | **Decompensated cirrhosis recipients (N=32)** | **P value** |
| Age (y) | 44(42-52.5) | 47(35.25-53.75) | 0.47 | 44(41.25-51.25) | 48.5(38-55) | 0.75 |
| Gender(M/F) | 17/6 | 38/12 | 0.99 | 15/5 | 23/9 | 0.99 |
| Donor age (y) | 52(37.5-55) | 49(34.25-56.75) | 0.75 | 52.5(45.75-55) | 49.5(38-57) | 0.73 |
| Donor BMI (kg/m^2^) | 23.67(22.35-24.35) | 23.05(22-24.175) | 0.50 | 23.885(22.5-24.575) | 23(21.75-24) | 0.15 |
| WIT (mins) | 22(18.5-24.5) | 19.5(15-24.75) | 0.37 | 22(18.75-24) | 19.5(15.75-24) | 0.31 |
| CIT (mins) | 337.5(269.5-428.75) | 352(293.25-491.75) | 0.38 | 343(274-450) | 352(293.75-424.25) | 0.52 |
| Anhepatic phase (mins) | 52(47.5-60) | 57(50.25-65) | 0.07 | 52.5(48.25-60) | 56(50.75-62.25) | 0.22 |
| RBC (U) | 6(4-8) | 6(4-10) | 0.86 | 5.5(4-8) | 5(3.75-10) | 0.84 |

WIT: warm ischemia time, CIT: cold ischemia time, AHT: anhepatic time, RBC: red blood cell.
