# Supplementary material for: Liver Transplantation in Acute-on-Chronic Liver Failure: Excellent Outcome and Difficult Posttransplant Course
Source: Front Surg. 2022 Jul 4;9:914611. doi: 10.3389/fsurg.2022.914611 (PMC9289224; doi:10.3389/fsurg.2022.914611)
Supplement: Supplementary file 3 [file Table_3_v1.docx]

Supplementary Table3: Propensity score matching of transplanted and non-transplanted ACLF grade2-3 patients.

|  | **Before Propensity score matching** | | | **After Propensity score matching** | | |
| --- | --- | --- | --- | --- | --- | --- |
| **Characteristics** | **Transplanted ACLF grade2-3 (N=18)** | **Non-transplanted ACLF grade2-3 (N=186)** | **P value** | **Transplanted ACLF grade2-3 (N=18)** | **Non-transplanted ACLF grade2-3 (N=69)** | **P value** |
| Age (y) | 44 (42.25-52.5) | 48 (40-57) | 0.73 | 44 (42.25-52.5) | 47 (39-57) | 0.98 |
| Gender(M/F) | 14/4 | 149/37 | 0.76 | 14/4 | 54/15 | 0.99 |
| AARC score | 8(8-9) | 9(8-9) | 0.06 | 8(8-9) | 8(8-9) | 0.39 |
| MELD score | 27(25-28.75) | 30(26-35) | 0.002 | 27(25-28.75) | 28(25-31) | 0.13 |
| **Characteristics** | **Transplanted ACLF grade2-3 (N=14)** | **Non-transplanted ACLF grade2-3 (N=162)** | **P value** | **Transplanted ACLF grade2-3 (N=14)** | **Non-transplanted ACLF grade2-3 (N=50)** | **P value** |
| Age (y) | 44(42.25-51) | 48(40-57.75) | 0.49 | 44(42.25-51) | 48.5(40.25-57) | 0.50 |
| Gender(M/F) | 11/3 | 126/36 | 0.99 | 11/3 | 39/11 | 0.99 |
| AARC score | 8(8-9) | 9(8-10) | 0.13 | 8(8-9) | 8(8-9) | 0.59 |
| MELD score | 27(25-27.75) | 30(26-35) | 0.005 | 27(25-27.75) | 27.5(26-31.75) | 0.14 |
